# Effective community engagement in one health research in Sub-Saharan Africa: a systematic review

**DOI:** 10.1186/s42522-024-00126-4

**Published:** 2025-01-15

**Authors:** Sidney Sangong, Farrukh Ishaque Saah, Luchuo Engelbert Bain

**Affiliations:** 1 ICAP Global Health, Columbia University, Columbia Mailman School of Public Health, Yaoundé, Cameroon; 2 Zentrum fur Medizin und Geselschaft, Department of Medical Anthropology, University of Freiburg, Freiburg, Germany; 3 Department of Population and Health, Faculty of Social Science, University of Cape Coast, Cape Coast, Ghana; 4 Public Health Emergency Operations Center, Department of Clinical and Public Health Services, Ministry of Health, Kigali, Rwanda; 5 Department of Psychology, Faculty of Humanities, University of Johannesburg, Auckland Park, Johannesburg, South Africa; 6 African Population and Health Research Center, APHRC, Nairobi, Kenya

**Keywords:** Community engagement, One health, Zoonotic diseases, Sub-Saharan Africa

## Abstract

**Background:**

The one health (OH) approach, linking human, animal, and environmental health, relies on effective community engagement (CE), education, stewardship, and effective regional and global partnerships. For real impact, communities should be at the centre of research agenda setting and program implementation. This review aimed at synthesizing empirical evidence on how communities are involved in one health research. Specifically, the review aimed at documenting the extent of community involvement in one health research, as well as to identify the barriers and facilitators to effective community engagement in one health research in sub Saharan Africa.

**Methods:**

The study was a systematic review conducted using the 2020 Preferred Reporting Items for Systematic Reviews and Meta-Analysis (PRISMA) guidelines. Empirical peer-reviewed research articles on community engagement in one health research published from January 2000 to September 2023 in English or French were retrieved from seven databases: MEDLINE, EMBASE, CINAHL, Cochrane Library, WHO Afro Library, the National Institute for Health Research, and African Journals Online databases. The extracted data from the included studies were analysed using a thematic synthesis approach.

**Results:**

The final review and synthesis included eight studies. The extent of CE in the one health research approach is quite limited. Two main best practices of CE in OH research were: 1) Awareness raising on OH research through social mobilization, rural outreach sensitization, and wide community assembly and 2) Building local capacity through community-based OH Training and Leadership workshops. The barriers to effective CE included: inadequate community research literacy levels, contextual disparities in CE, inadequate dissemination of research findings, language barriers and ineffective and uncoordinated stakeholder involvement.

**Conclusion:**

The review underscores the importance of effective CE in one health research. The best practices for CE in one health research are raising awareness and co-creation which should guide future initiatives. There are cultural, geographical, linguistic, and educational constraints that pose barriers to CE, requiring a more integrated and community-centric approach to one health research in SSA. An effective CE in one health research through this approach will ultimately lead to more effective responses and control of zoonotic disease outbreaks.

## Introduction

One health is defined as an integrated, unifying approach that aims to sustainably balance and optimize the health of humans, animals and ecosystems [41]. According to the One Health High-Level Expert Panel, the one health approach mobilizes multiple sectors, disciplines, and communities at various levels of society to work together to foster well-being and tackle threats to health and ecosystems while addressing the collective need for clean water, energy, and air, safe and nutritious food, taking action on climate change, and contributing to sustainable development [40]. The one health approach acknowledges the connections between human disease, environment and animals; however, these approaches are only successful with bottom-up community engagement, education and international collaborations [13]. Even though the one health concept is new for many, it has historically and currently, impacted lives on a global scale [13]. Acknowledging the links among people, animals and their shared environment, the one health principles became particularly pertinent in the onset of the COVID-19 pandemic, with the virus transitioning from its bat host to an unidentified species then finally to humans with significantly disastrous effect in many communities [13].

Community engagement as a whole is a strategic approach for collaborative working targeting a society’s community stakeholders for the building of solid relationships and enhancing communication, in order to attain specific goals for the society. The overall focus of Community Engagement (CE) in one health research is to enhance the community’s aptness in addressing specific health challenges while pointing out these health problems to major stakeholders in research within the community. The major cornerstones for community-oriented health research processes are authenticity and credibility [10]. Effective community engagement is gradually considered a crucial factor in health research. A rising burden in the engagement of communities in research has been expressed by community stakeholders and funders [2]. Both local and global institutions acknowledge the significance of CE in research. Nevertheless, the inequality in authority existing between the researchers and community participants causes the community members to take the backseat in the decision-making process [11], often resulting in the complete withdrawal of community members from studies owing to the lack of trust [1]. Moreover, the guidelines for CE are quite unclear, leading to the difficulties of implementation, evaluation, possibly resulting in missed opportunities, poor decision making and wastage of resources [18]. Community engagement in health research can be defined as a process that incorporates inputs from people (within a community) who the research outcome will impact and involves these people or groups as equal partners throughout the research process [43]. This involvement may include co-designing research questions to solve problems, making decisions, influencing policies and creating programs and interventions that affect their own lives [43].

Through community engagement, community participants are empowered to play an active role in their own health and partake in decisions and structures that impact health and well- being [42]. By so doing, intentional engagement efforts help to unpack and address local issues and opportunities, identify and implement more effective grounds-up solutions and leverage local resources and networks for sustaining health interventions and outcomes [42]. The process is also essential in strengthening trust and respect between stakeholders which is key to achieving positive and sustainable health outcomes [42].

The general importance of the one health approach in sub-Saharan Africa (SSA) is on a steady rise. According to the Global Burden of Disease reports, zoonotic infections in SSA account for an estimated 26% DALYS lost to infectious diseases and 10% of total DALYS lost [12]. This burden is expected to be on a steady rise as increasingly dense populations in SSA interact with wild life and ecosystems face consistent deterioration secondary to the rising deforestation for agricultural purposes and grazing [12]. This highly illustrates the undeniable relevance of one health approaches in order to effectively address the rising burden associated to zoonotic infections in SSA. Nevertheless, in order to successfully implement the one health approach, effective community engagement is an indispensable backbone. However, so far, there is no benchmark on what community engagement is and how much of it is essential for one health interventions to practically mitigate the burden of zoonotic diseases in SSA. Moreover, solid practical frameworks are required in SSA in order to effectively design and implement programs favoring the one health approach, health policies, legislations and research engaging communities.

In SSA, very little has been done in terms of prioritization of community engagement on one health research. Nevertheless, a community engagement-one health approach was applied to implement a training program aimed at advancing the development of diseases risk management and mitigation skills among agro-pastoralists living adjacent to conservation areas in South Africa [6]. This led to the successful implementation of risk management strategies by 98% of the community participants during a three-month follow up period and included improved personal and domestic hygiene practices as well as enhanced animal housing [6].

Concerning best practices to effective community engagement in health research in sub Saharan Africa, a few are quite remarkable which include culture-oriented community engagement strategies, community empowerment and involving key stakeholders at the onset of each health research [4]. On the other hand, the barriers to effective community engagement in sub-Saharan Africa vastly outweigh the best practices, these barriers include communication barriers (language barriers, misinformation, lack of understanding within the community of scientific concepts and literacy barrier), bad memories from previous research, prioritization inadequacies, cultural, political and religious barriers [4].

The overall relevance of the one health research concept is growing exponentially on a global scale, but particularly in SSA with the greatest burden of zoonotic infections according to the Global Burden of Disease, in addition to its great cultural diversity. Therefore, the one health research approach, if endorsed by SSA governments and implemented with effective community engagements would be a crucial resilience mechanism resulting in significant health benefits in multiple SSA countries. This review seeks to incorporate existing reports on how to engage communities in a one health approach from different SSA countries. The review specifically explored the rationale, extent, practices, and barriers of community engagement in one health research in the region. It then proposes a framework for effective community engagement in one health research in SSA.

## Methods

### Study design and search strategy

The 2020 Preferred Reporting Items for Systematic Reviews and Meta-Analysis (PRISMA) guidelines was employed [28]. The review afforded qualitative synthesis of details of multiple studies with the aim of proposing a framework for effective community engagement in one health research in SSA. In order to select suitable studies, we used a combination of key words and medical title terminologies like “community engagement” or “community participation” or “community involvement” or “community implication” or “community-based” in combination with the terms “one health”, “research” or “research approach” were used to search a total of seven databases, namely MEDLINE, EMBASE, CINAHL, Cochrane library, WHO afro library, African Journals Online databases and the national institute for health research. The search strategy for this review is illustrated detailly in Table 3 in Appendix. The search covered literature published between January 1, 2000 to September 30, 2023.

### Study selection

The inclusion criteria for this study were published studies in the domain of one health research and community engagement in SSA. All included studies were either published in the English or French languages. The inclusion criteria for this review were: Peer-reviewed publications, studies published in either the English or French languages, primary studies in SSA published since the year 2000. The exclusion criteria were: Reviews and editorials, letters to editors, personal narrations and views. By means of the eligibility criteria, the titles and abstracts of citations were screened prior to full texts assessments of selected articles for inclusion in this study. All citations retrieved from database searches were exported into Endnote 20 to get rid of duplicate citations before importing into Rayyan QCRI for screening.

### Outcome measure

The extent to which one health research incorporates community engagement in its approaches in order to foster well-being and address health threats to the ecosystem while simultaneously contributing to sustainable development.

### Quality of evidence

The quality assessment tool for studies of diverse design (QATSDD) was used to assess the quality of the selected studies. The critical assessment catalogues for qualitative [17] and mixed studies were used [3, 34]. These tools evaluated the analytical quality of included studies and determined the possibility of bias in their outline and method interpretation. The quality assessment was independently done by SS and FIS and where there was difference of opinions, LEB resolved this through a third assessment informed by consensus with all authors. Each research paper received a score based on each criterion, and from these, index scores were produced. The overall quality scores of the studies included were calculated as a percentage of the total anticipated scores (20 for qualitative studies and 48 for mixed methods studies). Percentage scores exceeding 50% were classified as high quality, while those equal to or less than 50% were considered low quality.

### Data extraction and analysis

The studies were examined carefully with the major themes summarized in an excel file. Data on the surname of the first author, year of publication, and specific themes on community engagement in one health were extracted by two of the authors. The data extraction tool contained information on the author(s), year of publication, study design, study country, and specific themes. The specific themes comprised of Rationale for CE in the One health research approach, Extent of CE in the One health research approach, Best practices in CE, and Barriers to CE in One health research. The review employed thematic synthesis analysis.

## Results

### Search results

**Fig. 1:**
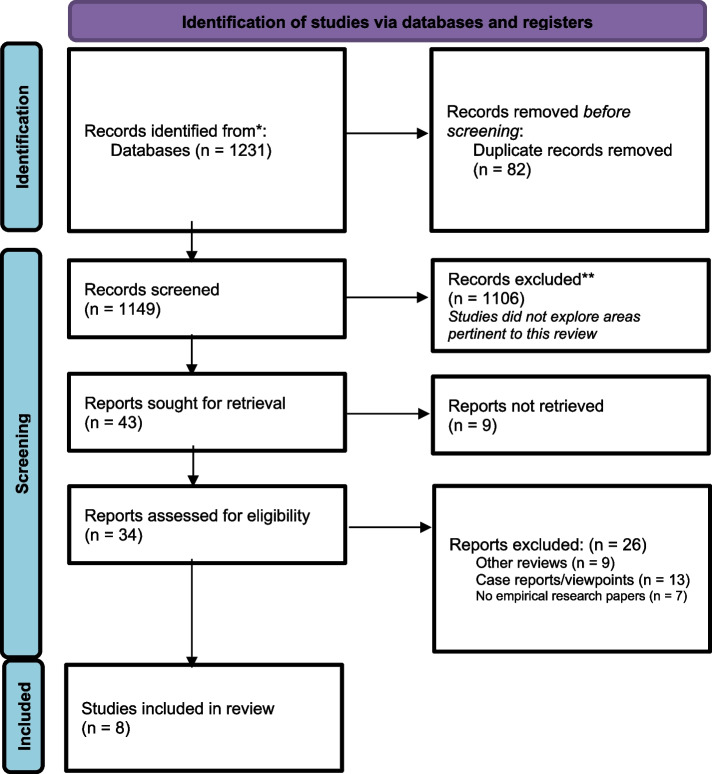
PRISMA flowchart for selected studies

Overall, 1231 studies were initially identified through search from the seven databases, more precisely: MEDLINE (679), EMBASE (173), CINAHL (104), Cochrane library (97), WHO afro library (51), African journals online databases (43) and the national institute for health research (84). After screening of the titles alongside their abstracts, 34 were retained. Following a full-text eligibility evaluation of these 34 studies, 8 met the inclusion criteria for our study. Figure 1 illustrates the PRISMA flowchart for the selection process of this review.

### Overview of included studies

All the eight studies in the provinces showed high quality ranging from lowest 70% [5] and 90% (Musesengwa et al. 2017) (Table 4 in Appendix). All included studies were conducted between 2016 and 2023. Eight of the included articles were qualitative studies based mostly on in-depth interviews and to a lesser degree, focused group discussions, meanwhile one of the included studies was a systematic review. Seven out of eight of the selected studies were single-country studies, including South Africa (3), Uganda (2), Nigeria (1), and Sierra Leone (1). One a multiple-country survey, this was conducted in Benin, Cote d’Ivoire, Burkina Faso, Cabo Verde, The Gambia, Ghana, Guinea, Guinea-Bissau, Liberia, Mali, Mauritania, Niger, Nigeria, Senegal, Sierra Leone, Togo, Burundi, Comoros, democratic Republic of Congo, Djibouti, Eritrea, Ethiopia, Kenya, Malawi, Mauritius, Mozambique, Mayotte, Madagascar, Reunion, Rwanda, Seychelles, South Sudan, Somalia, Sudan, Zimbabwe, Zambia, Uganda, Tanzania, Angola, Cameroon, Botswana, Central African Republic, Chad, Equatorial Guinea, Gabon, Republic of Congo, Sao Tome, and Principe and South Africa (Table 5 in Appendix).

Table 1 shows the thematic results of the review of the eight studies. One out of the eight articles selected for this review reported on the rationale of community engagement in one health research. Six of the eight included studies in this review examined the extent of community engagement in one health research. Additionally, six out of the eight included studies explored the best practices in CE. The barriers to effective community engagement in one health research in SSA were reported by three studies.

**Table 1:** Themes and Sub-themes of the qualitative review

**Rationale for Community Engagement in the One Health research approach**		
**Themes**	**Sub-themes**	**Research articles**
Risk mitigation strategies	Use of hands-on activities to train participants on infection control concepts	Berrian *et al *(2018) [6]
	Local capacity building and promotion of community acceptance of good health practices	Berrian *et al *(2018) [6]
	Use of community based One Health Training and Leadership (OHTL) workshops	Berrian *et al *(2018) [6]
**Extent of Community Engagement in One Health research**		
**Themes**	**Sub-themes**	**Research articles**
Multi-sectorial priority and ranking of zoonotic infections	Use of a facilitated consultative process involving community members in diverse domains of expertise including environmental, animal, human and agricultural professionals	Sekamatte *et al *(2018) [31]
Multi-sectorial community engagement for the mitigation of zoonotic disease transmission	Involving community members in the data collection process for desk reviews in the Arua and Moyo health districts	Medley *et al *(2021) [21]
	Use of facilitated discussions, primarily focused group discussions and key informant interview discussion guides	Medley *et al *(2021) [21] Musessengwa *et al *(2017) [24]
Rural community based participatory multicentre Ecohealth approach	Participatory Rural Appraisal (PRAs) Workshops	Musessengwa *et al *(2017) [24] Mthembu *et al *(2023) [23]
Community surveillance in outbreak	Use of social mobilization as a critical response Component	Bedson *et al *(2020) [5] Tambo et al (2018) [35]
	Use of community surveillance and rapid response approach practice coupled to pest Management	Tambo *et al *(2018) [35]
**Best Practices in Community involvement**		
**Themes**	**Sub-themes**	**Research articles**
Raise awareness on the One Health research approach	Social mobilization programs	Bedson *et al *(2020) [5] Tambo *et al *(2018) [35]
	Rural outreach sensitization campaigns	Musesengwa *et al *(2017) [24] Mthembu *et al *(2023) [23]
	Community implication in proposal development	Medley *et al *(2021) [21] Musesengwa *et al *(2017) [24]
Co-creation	Establishing community partnerships and coalitions	Berrian *et al *(2016)
	Community based One Health Training and Leadership (OHTL) workshop	Berrian *et al *(2016)
**Barriers to community engagement in One Health research**		
**Themes**	**Sub-themes**	**Research articles**
Cultural limitations	Skepticism against research organizations triggered by cultural beliefs	Musesengwa *et al *(2017) [24] Mthembu *et al *(2023) [23]
	Non-consenting community members against home visits	Berrian *et al *(2016)
Geographical limitations	Sparsely distributed communities	Berrian et al (2016)
	Inadequately constructed roadways	Berrian *et al *(2016)
Linguistic and Educational constraints	Inadequate community research literacy levels	Mthembu *et al *(2023) [23]
	Contextual disparities leading to difficult implementation and transferability of one health research procedures	Mthembu *et al *(2023) [23] Musesengwa *et al *(2017) [24]
	Inadequate dissemination of research findings secondary to discrepancies in vernacular languages.	Mthembu *et al *(2023) [23]

The rationale of community engagement in one health research included, implementation of risk mitigation strategies among community members and stakeholders against zoonosis and environmental traits to health, ameliorate domestic and personal hygiene practices as well as improved animal habitats. The extent of community engagement in the one health research approach was very limited in Sub Saharan African countries. Six out of the eight included studies reported on the extent of community engagement in the one health research approach. Only one of the included studies explored wider geographical locations and were multiple country surveys. The barriers and limitations to effective community engagement in one health research included: inadequate community research literacy levels, contextual disparities leading to difficult implementation and transferability of one health research procedures, inadequate dissemination of research findings secondary to discrepancies in vernacular languages and unsuccessful stakeholder involvement.

### Rationale for community engagement in one health research

Regarding the theme of risk mitigation, three sub themes were disclosed during our review including the use of hands-on community mobilization activities to train participants on infection control concepts, local capacity building and the promotion of community acceptance, and finally a community-based One Health training and Leadership (OHTL) workshops to foster infection control and tutor community members on one health research concepts. A single study in the review reported on the zoonotic infectious disease risk mitigation strategies [6]. There was equally a massive boost in the abilities of community participants to conduct an effective risk assessment [6]. Furthermore, concerning the local capacity building and promotion of community acceptance, including the OHTL workshops, an overall improved understanding of the disease prevention concepts and the one health approach was reported among community participants [6].

### Methods of community engagement

With regards to multisectoral priority setting and ranking of zoonoses as a measure of the extent of community engagement prioritization in one health research, just one sub theme was identified with one study exploring this [31]. This involved the use of a facilitated consultative process, involving community members in diverse domains of expertise including environmental, human, agricultural and vertinary professionals. This consultative process resorted to rank priority zoonotic diseases based on the recorded burdens in Uganda. Several criteria were taken into consideration for the scoring of zoonotic infections. These criteria included the severity of zoonoses in humans, availability of effective control strategies, potential to lead to an epidemic or pandemic in humans or animals, socioeconomic impacts and bioterrorism potential [31]. At the end of the consultative and priority setting exercise involving community actors, seven zoonotic diseases out of a total of forty-eight were identified as top priorities for Uganda which include anthrax, zoonotic influenza viruses, African trypanosomiasis, plague, rabies and viral hemorrhagic fevers [31]. This consultative process readily engaged communities and empowered its members by letting them get involved in every decision-making process to successfully have a hand in how to address these top infections, with prospects for future community engagement activities in effectively tackling zoonoses. Sensitivity analysis revealed no significant changes in the prioritization of zoonoses [31].

Another theme for the extent of community engagement in one health research is the multi-sectoral community mobilization for the mitigation of zoonotic disease transmission. Within this theme, the review found two sub themes which include involving community members in the data collection process for desk reviews in the Arua and Moyo districts and facilitated discussions precisely focused group discussions and key informant interview discussion guides. Two studies in the review examined these sub themes [21, 24]. In addition, the involvement of community members in the data collection process for desk reviews and highlighting zoonotic disease hotspots led to the implementation of an animal-adapted population mobility mapping, and showcasing the necessity of multisectoral initiatives in one health border approaches and research [21]. In addition, engaged communities, via focused group discussions and key informant interviews felt the continuous solicitation of their advice and preferences enabling them to considerably contribute to shaping the engagement process [24]. As a result, research naïve communities could significantly contribute to the research process when adequately engaged [24]. Even though these key informant interviews and focused group discussions were explored in both these studies, the overall impact post the activities mostly is felt by the ones involved in the activity, a greater proportion of the communities are very likely not to have a full grasp of all the decisions and knowledge sharing taking place during KII and FGDs hence the act of community engagement is not quite extensive enough.

Further themes pertaining to the extent of community engagement in one health research are the rural community-based participatory eco-health multicentre approach including two sub themes and finally community surveillance in outbreak with two sub themes as well. These two themes are covered by two studies in this review [23, 24]. As for the rural community based participatory eco-health multicentre approach, the two sub themes reported were participatory rural appraisal workshops in which community members participate as partners after significant community empowerment, participate in some capacity in geospatial disease and vector mapping for zoonotic disease control after identification of zoonotic infection hotspots [21, 23, 24]. With reference to community surveillance in outbreak response, two sub themes were as well identified in two studies [5, 35], these include the use of social mobilization programs as a critical response component and rapid response approach coupled to pest management also regarded as good practices in the one health CE approach.

### Common practices in community engagement in one health research

With respect to the best practices in community implication in one health research, seven studies in our review reported on the most convenient current practices for community engagement [5, 6, 13, 21, 23, 24, 35]. The first theme under this category entails raising awareness on one health research. This has three sub themes namely social mobilization, rural outreach sensitization and wide community assembly for proposal development. The social mobilization program sub theme was reported by two studies in the review [5, 35]. Social mobilization through large-scale participatory community engagement and real-time data collection reaching out to tenths of thousands of community participants via trained community mobilizers, mosques, churches and multiple local radio station announcements are necessary in emergency response and implementation of new disease control models as well as dissemination of research findings [5]. Moreover, social mobilization through advances in cloud sourcing and social media tools and solutions plays a significant role in effective CE by developing and integrating evidence-based timely risk communication and reporting systems in improving contextual community-based zoonotic disease control and immunization [35]. As a consequence, the research team uses these means to spread the research findings through an effective community engagement approach. Concerning rural outreach sensitization campaigns as a good practice in community engagement in one health research, two studies in the review reported on this sub theme [23, 24]. Rural outreach sensitization campaigns as a CE approach in research naïve societies promoted the empowerment communities through the constant solicitation of their opinions leading to a significant contribution of the research project [24].

Building the capacity of community volunteers on the research projects, and empowering certain community members as co-researchers on the research projects during the community based One Health Training and Leadership labelled under the theme of co-creation, could potentially play an important role in effective CE and addressing doubts regarding the research and the organizations involved, hence community members may tend to not feel left out of the research project. This was showcased by a single study as one of the best practices in community engagement in one health research [6]. These studies elaborated on the fact that local capacity building of community members and stakeholders on the one health research projects is imperative in tackling the research problem and regarded as an effective CE approach. Furthermore, community empowerment through these trainings and the OHTL workshop by training community facilitators demystifies the research projects and improves on the level of community engagement. The OHTL workshop hugely enabled the communication knowledge sharing between researchers and community stakeholders as well as mitigating condescension and counter-acting the inescapable power dynamics of this kind of scenario. As a result, the communities felt empowered which looks to be some of the best practices in one health research.

### Barriers to community engagement in one health research

Despite a few good practices in effective community engagement in one health research in SSA reported in the previous sub-section of this review, an effective CE process in OH has been explored by a rather very limited number of surveys included and leaves yet a lot to be desired in SSA. This could be explained by a plethora of reasons amongst which could be the fact that the one health concept is still new for many coupled to a significantly low science research gap experienced in Africa overall, with only a staggering twenty health researchers per million people being the least in world [39]. Besides these, there exist a good number of barriers to effective community engagement in one health research in SSA as reported in studies captured explained in this section of the review.

As regards cultural limitations, three themes were explored from three studies included in the review [6, 23, 24]. These include skepticism towards the research process triggered by cultural beliefs, non-consenting community members to home visits which are potential handicaps to a sound community engagement process. Skepticism and mistrust towards western educational methods and ideologies were significant setbacks to effective community engagement [24]. Hostility towards strangers led to the non-consenting behaviours of some community members to home visits which resulted in ineffective community engagement [23, 24]. Pertaining to the geographical constraints, sparsely distributed communities and inadequately constructed roadways were both hindrances to an effective community engagement [6]. Moreover, some community members expected hugely tangible benefits in the form of gifts and financial assistance as a form of motivation first in order to be involved community engagement activity [24] which was a drawback in the entire process.

Concerning linguistic and educational barriers, three sub themes were reported from two studies included in this review [23, 24]. This includes inadequate community literacy levels, contextual disparities leading to difficult implementation and transferability of one health research concepts and lastly inadequate dissemination of research findings secondary to discrepancies in vernacular languages. The limited knowledge and understanding of one health research concepts coupled to the low literacy levels of community members in rural areas significantly hindered effective community engagement. The contextual disparities and mentality on the part of the community participants believing the research and community engagement could be some sort of colonial exploitation led to the stressful implementation and transferability of one health research concepts to communities. Language barriers particularly on the part of the research team to comprehend the local languages resulted in inadequate community engagement and suboptimal dissemination of research findings.

## Discussion

Our review examined the rationale, extent, best practices and barriers to effective community engagement in one health research in sub-Saharan Africa. Overall, eight studies were reviewed to obtain the qualitative themes and sub-themes. One theme was found exploring the rationale for effective community engagement in one health research. This included the risk mitigation strategies against the transmission zoonoses. The methods of community engagement included multi-sectoral priority setting and ranking of zoonotic infections, multi-sectoral community engagement for the mitigation of the transmission of zoonoses, rural community- based participatory multicentre eco-health approach and community surveillance in outbreak. Raising awareness and co-creation were some of the best practices for community engagement in one health research while cultural, geographical, linguistic and educational constraints were the barriers to effective community engagement.

The development of evidence-based frameworks within the governance structure builds resilience to zoonoses and a plethora of environmental threats such as global warming and climate change, promoting the one health approach and potentially facilitating community involvement in one health research [26]. The development of evidence- based frameworks as explored in this review is in line with the establishment of the one health steering committee and workforce in Rwanda which mobilized multidisciplinary experts to prepare for potential one health research projects, coordinate responses and control zoonotic disease outbreaks [29, 38].

In our review, the use of hands-on training and capacity building workshops on the one health research strategy as an effective community engagement approach to mitigate the risk of transmission of zoonoses, proved to be beneficial in infection control and improved the knowledge of its community participants and agro-pastoralists adjacent to conservation areas in the Bushbuckridge local municipality Mpumalanga province in South Africa [6]. These findings are consistent with that of other studies in which capacity building workshops as an effective CE approach after the evaluation of existing surveillance systems and the initiation of multi-sectoral partnerships were crucial for identifying priorities and core themes of the one health surveillance systems in successfully attenuate the transmission of zoonoses with equitable inputs from various sectors [16, 32].

Multi-sectoral one health zoonotic disease prioritization workshops as a CE approach via a facilitated consultative process involving community members reported in this review resulted in the identification seven zoonotic diseases out of a total of forty-eight were identified as top priorities for Uganda which include anthrax, zoonotic influenza viruses, African trypanosomiasis, plague, rabies and viral hemorrhagic fevers [31]. This provides a great opportunity for the community involved to effectively prioritize while simultaneously strategizing on addressing zoonoses of the greatest concerns in check as well potentially improving health outcomes as whole. The role of multi-sectoral prioritization workshops in effective community engagement was also successfully explored in a study involving seven countries between the years 2014– 2017 [30].

Moreover, a multidisciplinary community engagement workshop as a CE strategy involving multiple one health stakeholders for the mitigation of the transmission of cross-border zoonoses in our review created a forum for sharing knowledge and learning about the one health research concept. The findings in our review are identical to a previous multiple-country study involving seven nations namely: Kenya, Thailand, Ethiopia, Azerbaijan, Cameroon, South Africa and the Democratic Republic of Congo. This multiple country survey led to a joint external evaluation involving vastly holistic members, that successfully incorporated multisectoral zoonotic disease prioritization with equal engagement from all sectors. More importantly, this led to the strengthening of one health partnerships for prioritized zoonoses and also put in place systems for the early detection and response to emerging diseases with a potential to become a global threat [30].

The application of a rural community-based participatory multicentre eco-health as a CE approach through participatory rural appraisal (PRAs) workshops proved that the research inexperience of communities should not be a hindrance to community involvement in one health research. This solely necessitates that the research teams and institutions should dynamically and carefully seek the community’s active contribution in the research process for knowledge sharing between researchers and community members. These findings are similar to others in Africa [7, 27, 36, 37] that have displayed the possibility for research naïve communities to effectively be involved in research despite the numerous setbacks that could initially interfere.

Co-creation as one of the best practices in effective community engagement in one health research in our review consisted of training community facilitators to play the role of panelists and co- researchers had a significant impact in addressing complexities as well as demystifying the research process. The co-creation in this review is consistent with the findings in previous studies in which community empowerment and capacity building remarkably impacted the research project as it bolstered the community’s possession and empowerment at the implementation phase [9, 14, 22, 25]. Raising community awareness on one health research through rural outreach sensitization campaigns and social mobilization programs were some of the best practices identified by research institutions in effectively engaging communities in one health research. The findings in our review were consistent with previous studies that highlighted the importance of community awareness creation in improving community implication in research [8, 20, 43].

In our review geographical limitations secondary to inadequately constructed roadways and sparsely distributed communities were barriers to effective community engagement in research. This is similar to the observations in other studies [19, 44]. In addition, cultural limitations resulting from skepticism against research organizations borne from cultural beliefs and non-consenting community members to research projects were barriers to smooth community engagement. This could be explained by poor experiences from previous community-based research projects and failure of these projects to achieve their objectives thus discouraging communities from engaging in potential present and future research projects. These observations are consistent with those of previous studies [15, 33].

### Potential framework for effective community engagement in one health research in SSA

The rising necessity for multi-sectoral research in order to tackle today’s challenging health and environmental issues is to be considered a top priority globally. Nevertheless, the importance of the one health approach to research is even more relevant in sub-Saharan Africa with zoonotic infections accounting for over 25% of the years of healthy life lost to infectious diseases, with this figure potentially rising as the increasingly dense populations in SSA are set to further interact with the animal world, justified by a deteriorating ecosystem secondary to deforestation and grazing [12]. There is a growing need for practical frameworks in SSA that effectively engage communities in designing and implementing one health research programs adopted by the local governments, research institutions and universities.

In this regard, by applying the community engagement Vancouver Costal Health framework, as well as the Bay et al’s conceptual framework, we proposed a framework (see Table 2) based on the components influencing community involvement in one health research projects. The framework seeks to elaborate on the different phases of effective community engagement in one health research. For this framework, four different phases of effective community engagement were explored. These include informing, consulting, involving and empowering the communities.

**Table 2:** Proposed engagement-empowerment framework

Phase of Community Engagement	Goal	Method of execution
Notify	To provide balanced and targeted information to community members on the aim of the specific one health research project in order to facilitate the understanding of key research problems faced and potential solutions	Target individuals and groups to ease the understanding of the research problem. Also target institutional to ease dissemination of research information
Consult	To acquire feedback and decisions from community members and stakeholders	Consult individual members of the community, groups and niches with respect to their understanding of the project
Involve	To work with the community during the entire research process, enabling that the community concerns are mastered	Involve community members and groups with respect to individual understanding of the research project
Empower	Placing the final decision-making in the hands of the community	Empowering both and groups based on their respective potentials to make the final decision at the end of the research project

Firstly, informing communities of the one health research approach is of great importance as it gives the members a global idea on what it entails. Providing the community members with this global idea includes steady and key details to assist in the understanding of the problems, potential solutions from a one health research approach and the overall benefits. This would include general details on the mode of transmission of vector-borne and zoonotic infections and how to prevent and control them. Equally, informing communities on how their environment/ecosystem directly or indirectly impacts their health in both positive and negative ways must be considered for better clarity on safety measures during the research process. Next, the community members should be tutored on the various one health research concepts and briefly trained on how to participate in the research project by effectively engaging community members in a holistic manner.

Secondly, the consultative process mostly requires obtaining feedback from communities on their various one health concerns. This includes listening to the community members carefully taking into consideration every one health experience obtained and applying these in the research to validate their participation and enabling them have a say in every decision-making process during the one health research project.

Thirdly, involving community members requires implicating them at every stage enabling the community and institutions are effectively understood throughout the project. This also is to endeavor the societal concerns and challenges are reflected in every procedure of the research process.

Finally, community empowerment necessitates entirely leaving the decision-making process with regards to the research findings in the hands of the community members and stakeholders. Table 2 below expounds on the key stages of the community empowerment process.

### Strengths and limitations

This review’s major strength is its inclusion of a diverse range of studies, both qualitative and mixed-methods. This provided a comprehensive and nuanced understanding of the subject matter. Secondly, the review follows a rigorous methodology, using standard tools to evaluate the analytical quality of the included studies and assess the potential for bias. This enhanced the reliability and validity of the findings. Lastly, the review adopts a thematic synthesis approach, which is particularly suited to synthesizing findings from qualitative and mixed- method studies, providing rich insights into effective community engagement in one health research.

Nevertheless, the review also has some limitations. While the majority of the studies were qualitative, the review may lack the quantitative data necessary to measure the impact or effectiveness of community engagement strategies. This points to a need for more quantitative and intervention studies in this area. Also, the findings may not be applicable to all contexts within the SSA region due to variations in cultural, geographical, and socio-economic factors. This could not be mitigated as there were not significant number of studies from the different countries in the region.

### Implications

This systematic review provides valuable insights and has several implications for the development of policies, strategies, and practices for effective community engagement in one health research in SSA. The findings of this review could guide future research in this area, contributing to the advancement of knowledge in this field. It also identifies areas that require further research, particularly in the development of strategies to overcome identified barriers and in the evaluation of the effectiveness of identified best practices. Firstly, it emphasizes the importance of risk mitigation strategies in addressing zoonotic infections via the use of local capacity building and community-based one health training and leadership workshops as CE approach. This aligns with the establishment of the one health steering committee and workforce in Rwanda, setting a precedent for other SSA countries to follow. Additionally, the findings emphasize on the significance of effective community engagement in mitigating the transmission of zoonoses, including other CE approaches in community-based priority setting and ranking of zoonotic infections, rural community-based participatory multicentre eco-health approaches, and community surveillance in outbreaks.

More so, the review highlights raising awareness and co-creation as best practices for effective community engagement in one health research. These practices could be incorporated into future community engagement strategies to enhance their effectiveness. However, the review also identifies cultural, geographical, linguistic, and educational constraints as barriers to community engagement. Recognizing these barriers is the first step towards developing strategies to overcome them, thereby improving community engagement in one health research.

## Conclusion

This systematic review has provided significant insights into the extent, rationale, best practices, and barriers to effective community engagement in one health research in sub-Saharan Africa. The implementation of effective risk mitigation strategies is a crucial step towards enhancing resilience to zoonotic infections. The review also underscores the importance of effective community engagement as an effective community engagement appraoch in mitigating the transmission of zoonoses, highlighting the need for priority setting, ranking of zoonotic infections, rural community-based participatory multicentre eco-health approaches, and community-based surveillance systems during outbreaks.

The identification of raising awareness and co-creation as best practices for effective community engagement in one health research provides a valuable guide for future initiatives. However, the review also brings to light the cultural, geographical, linguistic, and educational constraints that pose as barriers to effective community engagement. These findings highlight the need for strategies that address these barriers to enhance community engagement in one health research.

Overall, this review not only contributes to the existing body of knowledge but also provides a potential framework for community engagement in one health research in the region. It is hoped that these findings will guide future research and policy-making in this area, ultimately leading to more effective community engagement strategies that address control of zoonotic disease outbreaks and improves the overall health outcomes of communities. It thus serves as a stepping stone towards a more integrated and community-centric approach to one health research in sub-Saharan Africa.

## Data Availability

Data sharing is not applicable to this article as no datasets were generated or analyzed during the current study. Nevertheless, the selected articles have all been listed in Table 4 in Appendix.

## Appendix

**Table 3:** search strategy for Medline through OVID SP

**SN**	**Search Elements**
1.	Community Implication/ or Community-Based One Health Research approach/ or Community engagement.mp.
2.	((Engagement or engaging or participat* or involv* or implicat*) adj3 Communit*).m_titl.
3.	limit 2 to abstracts
4.	1 or 2 or 3
5.	benin/ or burkina faso/ or cape verde/ or cote d'ivoire/ or gambia/ or ghana/ or guinea/ or guinea-bissau/ or liberia/ or mali/ or mauritania/ or nigeria/ or senegal/ or sierra leone/ or togo/ or (africa*adj2 west* or benin* or burkina fas* or cape verd* or cabo verd* or ivory coast or cote d'ivoire* or gambia* or ghana* or (guinea* not pig*) or bissau or liberia* or (mali not fowl) or malian or mauritania* or nigeria* or senegal* or sierra leon* or togo* or (Lagos or Accra or Abidjan or Dakar or Abobo or Abuja or Freetown or Ouagadougou or Conakry or Lome or Bamako or Cotonou or Kumasi or Monrovia or Ibadan or Kano or Port harcourt or Benin City or Porto Novo or Niamey or Yamoussoukro or Banjul or Timbuktu or Djenne or Abomeyu or Zaria or Tamale or Jos or Cape Coast or Maidugul or Aba or Gao or Calabar or Warri or Maiduguri or Bobo Dioulasso or Parakou or Djougou or Bohicon or Sekondi Takoradior Sunyani or Obuasi or Teshie or Tema or Sikasso or Kalabankoro or Nouakchott or Dakhlet Nouadhibou or Benin City or Port Harcourt or Ilorin or Kaduna or Enugu or Ikorodu or Onitsha or Bauchi or Akure or Abeokuta or Sokoto or Bouake or Makeni or Kaduan or Sosgbo or Osogbo or! Gombe or Ilesa or Badagry or makurdi or Sagamu or Iseyin or obbomosho or Awka or Ado Ekiti or Nsukka or Ikeja or Katsina or Okene or Lafia or Minna or Ondo city or Umuahia or Calabar or Yola or Pikine or Touba or Thies Nones or Saint Louis or Kolak or Ziguinch or (San Pedro not (Spain or Mexico or Argentina or California or United States or Italy)) or Bandama or Daloa or Owerri or Kandi or Ifi or Dakar or Ogbomosho or Divo or Korhogo)).mp. [mp=title, abstract, original title, name of substance word, subject heading word, floating sub-heading word, keyword heading word, organism supplementary concept word, protocol supplementary concept word, rare disease supplementary concept word, unique identifier, synonyms]
6.	(angola or cameroon* or chad* or tchad or congo* or DRC or equatorial guinea* or gabon* or "Sao Tome" or Principe or Luanda or lobito or kuito or huambo or Malanje or Douala or Yaounde or Bamenda or Garoua of Bafoussam or Ngaoundere or Maroua or Kouosseri or Buena or Kumba or N'Djamena or Moundou or Bangui or Bimbo or Brazzaville or Point Noireor Kinshasa or Lubumbashi or Leopoldville or Elizabethville or Mbuji Mayi or Bakwanga or Bukavu or Costermansville or Kananga or Luluabourg or Kisangani or Stanleyville or Tshikapa or Koalwezi or Likasi or Jadotville or Goma or Kikwit or Uvira or Bunia or Mbandaka or Coquilhatville or Matadi or Butembo or Kabinda or Mwene Ditu or Isiro or Paulis or Boma or Kindu or Bata or Malabo or Libreville).mp. [mp=title, abstract, original title,name of substance word, subject heading word, floating sub-heading word, keyword heading
	word, organism supplementary concept word, protocol supplementary concept word, raredisease supplementary concept word, unique identifier, synonyms]
7.	((British Indian Ocean Territory or Burundi* or Comoros or Djibouti* or Eritrea* or Ethiopia* or Kenya* or Madagascar or Malawi or Mauritius or Mayotte or Mozambique or Reunion or Rwanda* or Seychelles or Somalia* or Sudan* or Tanzania* or Uganda* or Zambia or Zimbabwe or Crozet Islands or Iles Crozet or Scattered Islands ! or Iles E! parses or Mwanza or Zanzibar or Eldoret or Morogoro or Hargeysa or Berbera or Nyeri or Mbeya or Machakos orMarka or Tabora or Iringa or Gondar or Meru or Geita or Musoma or Mtwara or Songea or Kigoma or Dese or Mek'ele or Bahir Dar or Jimma or Sinyanga or Korogwe or Nairobi or "Dar es Salaam" or Mombasa or Addis Ababa or Kampala or Kigali or Mogadishu or Dodomoa or Bujumbura or Nakuru or Anananarivo or Kisumu or Maputo or Asmara or Lusaka or Harare orPort Louis or Arusha or kitale or lilongwe or malindi or machakos or hargeisa or Bulawayo or Ruiru or Lamu or Kire Dawa or Kikuyu or naivasha or mwanza or tanga or nanyuki or voi or garissa or lodwar of kakamega or maralal or kitui or webuye or Axum or Nyahururu or Jinja orKismayo or Namanga or Mumias or Moshi or Moroni or Lokichogio or Hola or Rwenzori Mountains or Lake Victoria or Puntland* or (Adiharush or Ali-Addeh or Alinjugur or Buramino or Dadaab or Dagahaley or Dollo Ado or Fugnido or Hagadera or Hilaweyn or Ifo orKakuma or Kambioos or Kayaka II or Kobe or Kyangwali Nakivale or Nyarugusu or Wad Sherife or Bokolmanyo or Melkadida or Rwamanja)) adj5 (camp or refug*)).mp. [mp=title, abstract, original title, name of substance word, subject heading word, floating sub-heading word, keyword heading word, organism supplementary concept word, protocol supplementaryconcept word, rare disease supplementary concept word, unique identifier, synonyms]
8.	a/ or botswana/ or lesotho/ or malawi/ or mozambique/ or namibia/ or south africa/ waziland/ or zambia/ or zimbabwe/ or ((africa* adj2 south*) or angola* or botswana* sotho* or malawi* or mozambiq* or namibia* or swaziland or zambia* or babwe or Zuluor Tsonga or Xhosa or Swazi or Ndebele or Tswana or Sotho or Shona e or BaLunda or Mbundu or Ovimbundu or Chaga or Sukuma or Pretoria or Cape n or Johannesburg or Durban or Port Elizabeth or Bloemfontein or Windhoek or eru or Pietermaritz or (Kimberley not Australia) or Nespruit or Soweto or kwane or Limpopo or Rustenburg or Mahikeng or Oudtshroom or! Stellenb! osch or l or Gaborone or Luanda or Cabinda or Huambo or Lubango or Kuit or Malanje or ito or Lilongwe or Blantyre or Mzuzu or Maputo or Matola or Beira or Nampula or moio or Nacala or Quelimane or Lusaka or Kitwe or Ndola or Kabwe or Copperbelt are or Bulawayo or Chitungwiza or Mutare or Masvingo or Monashonaland or icaland).mp. [mp=title, abstract, original title, name of substance word, subject ing word, floating sub-heading word, keyword heading word, organism lementary concept word, protocol supplementary concept word, rare disease supplementary concept word, unique identifier, synonyms]
9.	5 or 6 or 7 or 8
10.	4 and 9
11.	limit 10 to yr="2000 - 2023"

**Table 4:** Quality assessment of included studies

	Quality assessment item number and scores (Qatsdd)																
Author, year	1	2	3	4	5	6	7	8	9	10	11	12	13	14	15	16	Total % score
	*Qualitative Studies*^*1*^																
1. Berrian *et al *2018 [6]	2	2	2	2	1	2	1	1	2	2							85
2. Mthembu Z, Chimbari M. 2023 [23]	1	2	2	1	2	2	2	2	1	2							85
3. Berrian *et al *2016	1	2	1	2	2	1	2	1	2	2							80
4. Bedson et al. 2020 [5]	2	2	2	1	1	1	1	2	1	2							75
5. Musesengwa *et al. *2017 [24]	2	2	2	2	2	2	2	2	1	2							90
6. Medley *et al. *2021 [21]	2	1	2	1	2	1	2	2	2	2							85
	*Mixed Methods Studies*^*2*^																
7. Tambo *et**al*. 2018 [35]	3	2	3	2	2	3	3	2	3	2	2	2	3	2	2	3	81.2
8. Sekamatte *et**al. *2018 [31]	3	3	3	2	2	3	2	3	2	3	3	2	3	3	2	3	83.3
^1^N/A –Not applicable EX-Exclude				0-No				1-Unclear				2-Yes			IN-Include		
^2^N/A –Not applicableIN-Include				0-Not at allEX-Exclude				1-Very slightly				2-Moderately			3-Complete

**Table 5:** List of included studies

Authors	Year of Publication	Title	Site	Study Design/Procedure
1. Berrian AM, Smith MH, van Rooyen J, MartÃ•nez-LÃ³pez B, Plank MN, Smith WA, Conrad PA.	2017	A community-based One Health education program for disease risk mitigation at the human-animal interface	South Africa	Qualitative study design (Focused group discussions)
2. Bedson J, Jalloh MF, Pedi D, Bah S, Owen K, Oniba A, Sangarie M, Fofanah JS, Jalloh MB, Sengeh P, Skrip L, Althouse BM, HÃ©bert- Dufresne L.	2020	Community engagement in outbreak response: lessons from the 2014-2016 Ebola outbreak in Sierra Leone	Sierra Leone	Community engagement in zoonotic outbreak response
3. Berrian AM, van Rooyen J, MartÃ•nez-LÃ³pez B, Knobel D, Simpson GJ, Wilkes MS, Conrad PA.	2016	One Health profile of a community at the wildlife- domestic animal interface, Mpumalanga, South Africa	South Africa	Ethnographic qualitative study design
4. Mthembu Z, Chimbari M.	2023	Community engagement: health research through informing, consultation, involving and empowerment in Ingwavuma community	South Africa	Qualitative study design
5. Tambo E, Adetunde OT, Olalubi OA.	2018	Re-emerging Lassa fever outbreaks in Nigeria: Re-enforcing "One Health" community surveillance and emergency response practice	Nigeria	Mixed methodology, including descriptive, analytical, qualitative, cross- sectional, and experimental design
6. Musesengwa R, Chimbari MJ.	2017	Experiences of community members and researchers on community engagement in an Eco-health project in South Africa and Zimbabwe	South Africa and Zambia	Qualitative Case study Approach
7. Medley AM, Gasanani J, Nyolimati CA, McIntyre E, Ward S, Okuyo B, Kabiito D, Bender C, Jafari Z, LaMorde M, Babigumira PA, Nakiire L, Agwang C, Merrill R, Ndumu D, Doris K.	2021	Preventing the cross-border spread of zoonotic diseases: Multi- sectoral community engagement to characterize animal mobility- Uganda, 2020	Uganda	Qualitative study design (Focused group discussions)
8. Sekamatte M, Krishnasamy V, Bulage L, Kihembo C, Nantima N, Monje F, Ndumu D, Sentumbwe J, Mbolanyi B, Aruho R, Kaboyo W, Mutonga D, Basler C, Paige S, BartonBehravesh C.	2018	Multi-sectoral prioritization of zoonotic diseases in Uganda, 2017: A One Health perspective	Uganda	Semi-Quantitative survey
